# A Paradigm Shift in Microbial Protein Manufacturing

**DOI:** 10.3390/life16010129

**Published:** 2026-01-14

**Authors:** Xinyu Zhuo, Yanzi Xie, Jiali Yu, Wandi Xue, Yijie Weng, Sheng Tong

**Affiliations:** School of Life Sciences, Southwest University, Chongqing 400715, China

**Keywords:** microbial protein, texture engineering, structural mimicry, bacterial cellulose, synthetic biology

## Abstract

Against the backdrop of the global protein crisis and the textural limitations of alternative proteins, microorganisms are increasingly recognized as versatile structural materials to address these challenges. This review systematically analyzes three key microbial strategies: employing mycelial solid-state fermentation to engineer fibrous meat analogues; utilizing bacterial cellulose scaffolds to enhance the texture of both cultured meat and plant-based products; and applying synthetic biology to design tailored functional proteins. Existing studies confirm that mycelial fermentation significantly improves product texture and production sustainability. In parallel, bacterial cellulose provides highly biocompatible nanoscaffolds, while synthetic biology enables the efficient production and nutritional enhancement of complex animal proteins. Although challenges in scaling production and optimizing flavor persist, advanced bioprocess optimization and genetic engineering offer promising solutions. Future breakthroughs are expected to transition from structural mimicry to true functional creation, establish decentralized production networks, and advance dynamic 4D-printed foods, which will collectively contribute to a more sustainable and resilient global food system.

## 1. Introduction

The dual pressures of continuous global population growth and ongoing consumption upgrades are placing unprecedented strain on traditional animal protein production systems, exposing their inherent resource-intensive and environmentally costly nature. The global meat consumption, which reached 328 million tons in 2021, is expected to increase by about 70% by 2050, and the existing livestock industry, which utilizes limited resources, is having difficulty meeting the demand [1]. Consequently, developing sustainable alternative proteins has become an urgent priority for the food industry. However, mainstream alternative proteins (such as plant-based meat) still face a core bottleneck on the path toward widespread acceptance: sensory attributes, particularly the difficulty in replicating the complex, fibrous texture of real meat [2]. Against this backdrop, microbial protein (or single-cell protein) demonstrates significant potential due to its high resource conversion efficiency and climate-resilient production models [3]. This paper aims to highlight that the value of microorganisms extends far beyond serving as a “protein source”; a more disruptive paradigm is emerging—viewing them as “living structural materials” to fundamentally address the challenge of texture simulation.

The necessity of this new paradigm becomes particularly clear when examining the limitations of existing texturization technologies. Current mainstream structuring technologies, such as high-moisture extrusion, can form basic protein networks but struggle to accurately replicate the complex microstructure of real meat, consequently resulting in final products with inferior mouthfeel and juiciness [4,5]. While technologies such as 3D printing allow for texture customization, their use for the precise structuring of plant proteins is nevertheless still in its infancy. Therefore, the industry urgently requires a novel approach capable of fundamentally constructing complex biomimetic structures. This is precisely where the paradigm of “microbial living structural materials” begins to demonstrate its disruptive potential. The ability to move beyond existing technological limits and create complex biomimetic textures from the ground up is pivotal for the next stage of growth in the alternative protein sector [2].

This paradigm fundamentally shifts the logic of utilizing microorganisms. Traditionally, the utilization of microorganisms has been largely confined to their metabolic products, primarily regarding them as “cell factories” for producing antibiotics, enzymes, or food ingredients. However, an emerging paradigm recasts the dynamic behaviors of microorganisms, including mycelial growth, biofilm formation, and polymer secretion, as a novel and powerful texture-engineering mechanism. The core advantages of this paradigm lie in three key dimensions. From a sustainability perspective, microorganisms can be cultivated on low-cost, renewable substrates such as industrial and agricultural waste, enabling a low-energy “waste-to-value” conversion with a minimal environmental footprint. For instance, the filamentous fungus *Neurospora intermedia* can be bioconverted on plant residues (thin stillage) and waste bread into additional ethanol, biomass, and pigment-rich feed products [6]. Second, microorganisms demonstrate significant structural self-assembly capabilities. A prime example is mycelia, which sense, directionally grow, and self-assemble into complex 3D network structures by interweaving [7]. Bacterial cellulose (BC) produced by *Komagataeibacter xylinus* possesses excellent mechanical and barrier properties, water-holding capacity, and structural integrity, attributed to its three-dimensional nanofiber network [8]. Regarding programmability, synthetic biology provides the tools for precise genetic programming of microbial strains. Advances in multiplex genome editing of microbes are set to revolutionize the engineering of advanced cell factories, offering unprecedented efficiency [9]. Building on this, the present review aims to systematically explore how these properties of microorganisms can be translated into structured solutions, assess their scalability challenges, and envision ways to truly unlock the full design potential of biology for constructing next-generation alternative proteins.

## 2. The Unique Advantages of Microbes as Living Structural Materials

“Living structural materials” are defined as engineered microbial systems characterized by autonomous structure formation, dynamic environmental responsiveness, and functional customization. The structural self-assembly, superior responsiveness, and robust programmability inherent in these systems form the theoretical foundation for three key technical pathways to build alternative protein structures.

### 2.1. Inherent Structural Shaping Capacity

Microorganisms, particularly fungi (such as *Lentinus edodes* and *Aspergillus* spp.) and certain bacteria (e.g., *Myxobacteria* and *Streptomyces* spp.), exhibit inherent growth patterns that enable autonomous formation of intricate microstructures. The dense, interwoven network of fungal hyphae imparts crucial structural attributes, including moisture retention and meat-like texture, which are vital for product formulation [10]. Bacterial cellulose (BC), a microbially synthesized extracellular biopolymer, possesses exceptional properties dominated by its ultra-fine nanofiber network that confers high porosity, water retention, and transparency [11]. For instance, BC produced through *Komagataeibacter* fermentation exhibits outstanding hydrophilicity, characterized by high water retention and swelling capacity [12]. This nanostructured network enables superior hydration, gelation, and stability, allowing it to replicate the lubricating sensation of fats or serve as a scaffold for encapsulating flavor compounds and nutrients. The structural repertoire of microorganisms extends beyond BC. Prokaryotes, notably bacteria, efficiently produce diverse extracellular polymeric substances (EPS). These biopolymers, such as commercial xanthan gum or gellan, serve as excellent viscosity modifiers and gelling agents, imparting desired rheological properties to food matrices and enhancing moisture retention and isotropic texture [13]. In contrast, eukaryotic fungi leverage their multicellular, filamentous growth to form expansive mycelial networks. This inherent macro-scale architecture closely mimics the fibrous, anisotropic structure of animal muscle, providing the primary structural skeleton for meat-like bite and mouthfeel [14]. Thus, bacterial EPS are ideal for fine-tuning matrix properties, while fungal mycelia are unparalleled for creating macro-structural foundations. This ability to spontaneously form complex microstructures through growth and secretion, which is unmatched by any non-living materials or conventional processing techniques, thus lays a biological foundation for constructing meat-like textures.

### 2.2. Environmental Sustainability and Circular Economy Potential

Life-cycle assessment demonstrates that microbial protein (MP), or single-cell protein (SCP), is substantially more resource-efficient than conventional animal agriculture. Its cultivation occurs in tightly controlled bioreactors, is largely decoupled from geographical and climatic constraints, and represents a promising sustainable pathway for future food production [15]. Moreover, microbial protein production is fundamentally arable-land-free and can convert low-value feedstocks, such as methanol, CO_2_, and lignocellulose from industrial and agricultural waste, into carbon and nutrients, aligning with circular bioeconomy principles. A prime example is found in methylotrophic organisms, which inherently utilize methanol as their sole carbon and energy source; engineering such bacteria into cellular production platforms thus offers an economically and environmentally sustainable approach [16]. Beyond gaseous and liquid substrates, filamentous fungi excel at valorizing solid agricultural residues. A prominent example is Neurospora intermedia. It can be cultivated on oat hulls, wheat bran, or other lignocellulosic by-products via solid-state fermentation to produce mycoprotein. This protein offers a complete amino acid profile and is rich in potassium, iron, and fiber, making it an appealing ingredient for innovative meat-alternative products [17]. Therefore, utilizing microorganisms goes beyond mere protein production to encompass a systematic solution for waste valorization and low-carbon production, which closely aligns with the sustainability goals of future food systems.

### 2.3. High Programmability Enabled by Synthetic Biology

Synthetic biology provides a powerful toolkit for the precise design and reprogramming of microbial systems [18]. Enabled by technologies such as CRISPR/Cas9, researchers can now perform precision genome editing in industrial microbial strains. For instance, Ferrando et al. demonstrated chromosomal integration of multiple amyQ gene copies in *Bacillus subtilis* using CRISPR/Cas9, significantly enhancing α-amylase activity [19]. Beyond genome editing, well-established expression systems like *Escherichia coli* remain invaluable for recombinant protein production due to their rapid growth, cost-effectiveness, scalability, and tunable expression levels [20]. For a wild-type Aspergillus aculeatus strain, Christina S Nødvig et al. have used CRISPR Cas9 system to generate a strain that contains an *AACU_pyrG* marker and demonstrated that the resulting strain can be used for iterative gene targeting [21]. The potential of these synthetic biology approaches is further illustrated by a 25-fold increase in taxadiene titer achieved in *Saccharomyces cerevisiae* through CRISPR/Cas9-mediated engineering and TXS-MBP fusion protein expression [22], showcasing the transformative impact of synthetic biology on customizing microbial functions. This implies that we can not only harness their innate properties but also “program” them-genetically engineering their structural formation, metabolic output, and other traits to achieve a leap from “utilizing nature” to “designing life”.

In summary, microorganisms have established the theoretical basis for their role as next-generation food “structural engineers,” owing to their inherent structure-forming ability, exceptional sustainability, and high degree of programmability. The core technology for achieving this, synthetic biology and metabolic engineering, has been amply demonstrated through the precise genetic programming and production optimization of microorganisms. For example, in chemical and pharmaceutical production, conventional breeding has increased the penicillin yield of *Penicillium chrysogenum* by over ten-thousand-fold; *Saccharomyces cerevisiae* produces artemisinic acid, a precursor to antimalarial drugs; and *Escherichia coli* is used to efficiently synthesize compounds like 1,3-propanediol [23]. These examples underscore the potential of metabolic engineering to integrate microbial rapid growth with target product synthesis. Therefore, building on these proven pathways holds promise for engineering microorganisms into efficient cellular factories for fabricating food textures

## 3. Three Core Strategies for Microbial Construction of Alternative Protein Texture

Building on these advantages, both industry and academia have developed three primary technological pathways. These are not mutually exclusive but rather represent a spectrum of increasing sophistication-from leveraging natural structures to achieving precise artificial design. As “living structural materials”, microorganisms are advancing next-generation strategies for building alternative protein textures, marking a shift from leveraging natural structures to implementing precision artificial design. Currently, three core strategies have emerged and are evolving in parallel, collectively driving progress in this field.

### 3.1. Mycelium-Based Solid-State Fermentation: The Prevailing Technology

As the most mature technological platform, mycelium-based solid-state fermentation utilizes the intricate, three-dimensional network of filamentous fungi cultivated on solid substrates, providing an ideal biological scaffold that replicates the texture and chewiness of muscle fibers [24].

The key advantage of this approach lies in its superior sustainability and cost-effectiveness. Unlike liquid-state fermentation, which requires substantial water and energy inputs for agitation and aeration, solid-state fermentation employs agricultural by-products such as wheat bran and rice husk as substrates. This method reduces water consumption by 50–70% and energy costs by over 30%, while the low-moisture environment enhances process robustness [25]. *Neurospora intermedia* is a filamentous fungus commonly found in nature as a saprophytic organism, thriving on burned vegetation and contributing to the decomposition of charred plant matter. *N. intermedia* and *R. oryzae* can increase the protein concentration of oat hulls through solid-state fermentation, demonstrating a way to convert a lignocellulosic-rich by-product, oat hulls, into a novel food [17]. Commercial viability is demonstrated by the sustained success of industry pioneer Quorn (UK). Since the 1960s, Quorn has utilized *Fusarium venenatum* for meat alternative production and maintains nearly 40% of the UK market share, with 2022 sales reaching £228 million, confirming strong market acceptance [26]. Meanwhile, research continues to expand application boundaries. A study has shown that incorporating merely 5% fungal protein from *Penicillium* solid-state fermentation significantly improved fibrous structure, chewiness, and gel properties of pea-based meat analogues while enhancing in vitro protein digestibility [27]. As the technology currently closest to scalable commercial application, mycelium solid-state fermentation has validated the feasibility of microorganisms as structural materials and provided the industry with valuable production and market experience.

### 3.2. Bacterial Nanocellulose Scaffolding: The Bridge for Cross-Application

Bacterial cellulose (BC), particularly the bacterial nanocellulose (BNC) produced by *Komagataeibacter* species, serves a dual role in the alternative protein industry owing to its unique nanoscale 3D fibrous architecture: it functions as an ideal scaffold for cultured meat and as an effective texture enhancer for plant-based products [28]. Recent perspectives highlight BC as a key sustainable biomaterial with immense potential for creating next-generation food structures, owing to its biocompatibility, biodegradability, and tunable properties [29]. These advantages originate from the biosynthetic mechanism of BNC. Its self-assembled, high-purity, crystalline nanofiber network confers exceptional water-holding capacity (retaining up to 200 times its own weight), high mechanical strength, and structural mimicry of the native extracellular matrix, combined with GRAS (Generally Recognized as Safe) status and biocompatibility [30]. In cultured meat production, BNC-based three-dimensional scaffolds closely recapitulate the in vivo microenvironment, facilitating muscle cell adhesion, proliferation, and differentiation. When combined with biomaterials such as chitosan or gelatin, BNC forms composite scaffolds that guide organized cellular assembly and promote the development of muscle-like tissue [31]. In plant-based applications, BNC acts as a texture modifier that integrates efficiently into protein matrices. Its nanofibrillar network promotes the formation of stable composite gels with plant proteins, significantly improving gel strength, elasticity, and chewiness. Moreover, the high specific surface area of BNC enables effective entrapment of water and lipids, which enhances moisture and fat retention, reduces losses during cooking and storage, and improves perceived juiciness. This functionality has been successfully illustrated in low-fat meat and fish analogs, where BNC helps maintain desirable structure and flavor while reducing fat content [32]. Beyond texture, advanced BC-based composites are being engineered to actively improve nutritional delivery and shelf-life, broadening their utility in functional foods [33]. Recent advances in synthetic biology have further enabled precise control over BC production. Standardized genetic toolkits now allow programmable engineering of BC-producing strains, facilitating tailored modulation of cellulose yield, macromolecular morphology, and material properties. This capability marks a paradigm shift in the field, from structural biomimicry toward deliberate molecular creation [34].

The successful application of bacterial cellulose (BC) demonstrates that microorganisms can not only provide an overall texture but also serve as a key functional ingredient to precisely improve specific attributes (such as water-holding capacity and gel strength) of other protein-based products, showcasing powerful cross-sector integration capabilities.

### 3.3. Precision Design Based on Synthetic Biology: The Core Driver for the Future

Precision design based on synthetic biology marks a fundamental paradigm shift in microbial engineering. This approach aims to reprogram microorganisms into highly programmable “cellular factories” through rational design and reconstruction of metabolic networks, thereby enabling the tailored production of complex proteins and even entirely novel food ingredients [35]. This technical approach employs a methodology that leverages gene-editing tools such as CRISPR/Cas9, coupled with systems metabolic engineering, for the precise optimization and reconfiguration of microbial metabolism [36,37].

*Pichia pastoris*, for example, is established as a leading chassis organism due to its strong secretory protein production and capacity to grow on non-food carbon sources like methanol, facilitated by an increasingly sophisticated genetic toolkit comprising strong promoters and efficient signal peptides [38]. This approach has already delivered impactful applications. A carbon-nitrogen synergy strategy boosted methanol-to-SCP conversion efficiency in *P. pastoris* to 92% of the theoretical limit, revealing considerable economic promise [37]. A complementary breakthrough came with the engineering of *E. coli* for efficient methanol assimilation, creating a new pathway for the use of liquid non-food feedstocks [39].

Synthetic biology enables de novo production of complex animal proteins, including phosphorylated casein, in microbial hosts such as *E. coli* by mimicking natural modification pathways. Meanwhile, engineering classical systems like *Bacillus subtilis* through genome editing and secretion optimization has elevated their capacity to produce food-grade recombinant proteins, revitalizing these proven cell factories [40]. In cutting-edge research, artificial intelligence is transforming protein design from empirical guesswork into data-driven predictive design. Machine learning models predict protein functionality (e.g., gelation, emulsification) directly from sequence data, facilitating the computational screening or de novo design of superior functional proteins [27]. Simultaneously, directed evolution combined with high-throughput screening rapidly generates systematically optimized enzyme variants with higher catalytic efficiency for tailoring texture and flavor [41].

**Figure 1:**
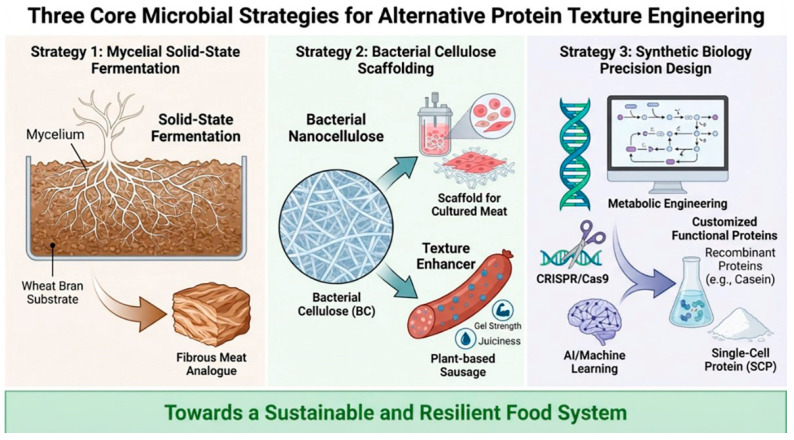
Microbial strategies for engineering alternative protein textures. This schematic illustrates three core approaches: (1) Mycelial solid-state fermentation utilizing fungal mycelium grown on wheat bran to form fibrous meat analogues; (2) bacterial nanocellulose scaffolding, which provides structural support for cultured meat and acts as a texture enhancer in plant-based products such as sausages; and (3) synthetic biology-enabled precision design, employing metabolic engineering, recombinant protein production (e.g., casein), and computational/AI-assisted modeling to tailor functional proteins and single-cell protein (SCP). Together, these strategies advance the development of sustainable and resilient food systems.

From the established practice of mycelium fermentation, to the versatile applications of bacterial cellulose, and further to synthetic biology design, these three pathways collectively form the technological blueprint for utilizing microorganisms as “living structural materials.” However, translating these laboratory or pilot-scale achievements into the broader consumer market still requires overcoming a series of practical challenges ( Figure 1 ).

### 3.4. Towards a Comparative Framework for Microbial Texturization Strategies

Despite the distinct advantages of the three core strategies, progress in the field is hindered by the lack of a systematic, quantitative comparison of their textural properties, resource efficiency, and scalability. Establishing a unified evaluation framework is indispensable for future research. Such a framework should integrate several critical dimensions: first, it must quantify mechanical and rheological properties—including tensile strength, elasticity, chewiness, and water-holding capacity—using standardized methods. Second, systematic sensory analysis conducted by trained panels is essential to assess attributes such as mouthfeel, juiciness, and overall likeness to animal meat. Methodologies from systematic reviews of analogous alternative protein products can provide valuable guidance for designing robust sensory evaluation protocols [42]. Concurrently, a comprehensive Life Cycle Assessment (LCA) should compare environmental impacts across the production cycle, encompassing energy consumption, greenhouse gas emissions, water use, and land footprint. Innovative approaches that integrate nutritional value with environmental impact assessment offer a promising direction for developing more holistic sustainability metrics for food systems [43]. Finally, the framework ought to incorporate key economic indicators, such as unit production costs at scale, yield efficiency, and capital investment requirements, to fully gauge commercial viability.

The development of such a multi-criteria framework will facilitate objective benchmarking, guide technology selection for specific applications, and accelerate the optimization and commercialization of microbial structure engineering approaches.

## 4. Real-World Challenges and Feasible Pathways

### 4.1. Scale-Up Control and Process Optimization

The large-scale cultivation of microorganisms in industrial-scale bioreactors commonly faces challenges such as heterogeneous cell growth, low metabolic efficiency, and difficulties in product quality control. The construction of enzyme-constrained models enables the precise prediction and regulation of metabolic flux in *Schizochytrium* sp., thereby significantly enhancing the synthesis efficiency of eicosapentaenoic acid (EPA) [44]. At the process level, multi-omics analyses have revealed the metabolic division of labor and collaborative mechanisms within microbial communities during the fermentation of Nongxiang Daqu, providing a theoretical basis for the functional regulation of microbial consortia in scaled-up cultivation [45]. Furthermore, strategies to enhance vitamin B12 fermentation stability include optimizing the carbon-to-nitrogen ratio and implementing dynamic control of key cofactors; this approach has been validated for industrial feasibility via patent documentation.

### 4.2. Flavor Modulation and Safety Compliance

Consumer acceptance of microbial protein is significantly influenced by its endogenous off-flavors and nutritional composition. Regarding flavor modulation, metabolic engineering approaches have enabled the de novo synthesis of key flavor compounds, such as medium-chain γ- and δ-lactones, in microorganisms, effectively improving the product’s flavor profile [46]. For nutritional enhancement, the metabolic reprogramming of industrial *S. cerevisiae* to reinforce its heme biosynthesis pathway has enabled high-efficiency heme production [47]. Similarly, low-carbon microbial manufacturing strategies based on yeast metabolic reconstruction can also facilitate the efficient synthesis of customized starch microparticles, demonstrating the potential of metabolic engineering in diversified product development [48]. The de novo biosynthesis of vitamin B12 in *E. coli* further demonstrates the application prospects of microbial cell factories in nutritional fortification [46]. Additionally, the integration of metagenomics and metabolomics methods allows for the systematic analysis of the formation patterns of flavor metabolites in multi-strain composite fermented douchi, providing theoretical guidance for flavor regulation [49].

Therefore, the future development of microbial protein products must adopt a “dual-focus” systems engineering approach that equally prioritizes both “technology” and “sensory attributes”. This entails actively shaping their flavor and nutritional profiles through tools such as metabolic engineering while also ensuring compliance with regulatory requirements via rigorous safety assessments. Additionally, beyond off-flavors, safety assessments must specifically address potential allergenicity and digestive tolerance of novel microbial proteins. Strategies such as in silico epitope screening, targeted hydrolysis to reduce allergenicity, and processing methods to break down indigestible cell walls are being explored to mitigate these risks [50].

### 4.3. Nutritional Equivalence and Safety Assurance

Beyond flavor, the successful integration of microbial proteins into mainstream diets hinges on achieving nutritional equivalence and ensuring safety. While many microbial proteins are complete, their amino acid profiles can differ from animal benchmarks. For instance, some fungal proteins may have lower levels of sulfur-containing amino acids like methionine, necessitating strategic blending or fortification in final products to meet nutritional standards [51]. Safety assessment is paramount, particularly regarding allergenicity. Proteins from novel microbial sources (e.g., mycoproteins from fungi) may pose risks to individuals with pre-existing allergies, and residual bacterial components (e.g., endotoxins) require stringent control during production [50]. Additionally, the presence of indigestible cell wall components (e.g., chitin, β-glucans) can affect protein bioavailability and cause gastrointestinal discomfort if not adequately processed. Addressing these challenges requires a multifaceted approach: utilizing bioinformatics for early allergenicity screening, optimizing fermentation and downstream processing to remove unwanted components and improve digestibility, and establishing clear regulatory frameworks and labeling guidelines for novel microbial proteins.

## 5. Paradigm Breakthroughs and Forward-Looking Perspectives

### 5.1. From Structural Biomimicry to Functional Creation: Designing Smart Foods Beyond Natural Meat

As the industry has matured, its strategic emphasis has shifted from primarily mimicking meat’s sensory properties toward a more integrated focus on product safety, biocompatibility, and nutritional adequacy. Mycelium represents an ideal platform for industrial meat alternative production, owing to its meat-like texture, rapid protein production rate, and low carbon footprint. The technological origins date to the 1970s collaboration between Rank Hovis McDougall and Imperial Chemical Industries, which pioneered early meat analogues using the fungus *F. venenatum*. Their method blended mycelium derived from soil isolates with processed biomass and binders, applying sequential steaming, freezing, and chilling steps to replicate meat-like structure [52,53]. Over the past decade, the sector has undergone remarkable expansion, with at least 51 companies globally now dedicated to fermented alternative proteins, more than 20 of which maintain product pipelines specifically centered on filamentous fungal fermentation.

There is growing consumer demand for meat analogues that are enriched with health-promoting factors such as omega-3 fatty acids and vitamins, offering nutritional profiles that may exceed those of conventional meat. For instance, Shahid et al. demonstrated that fungal protein intake improves acute postprandial glucose control and reduces circulating cholesterol concentrations [54,55]. For omega-3 fatty acids, essential nutrients that humans cannot synthesize, fungal platforms offer distinct advantages, including the ability to grow on diverse carbon sources, high cost-effectiveness, and scalability through genetic engineering [56]. The established combination of CRISPR/Cas9-mediated gene editing and fermentation processes paves the way for the industrial-scale production of these nutritionally essential omega-3 fatty acids.

**Figure 2:**
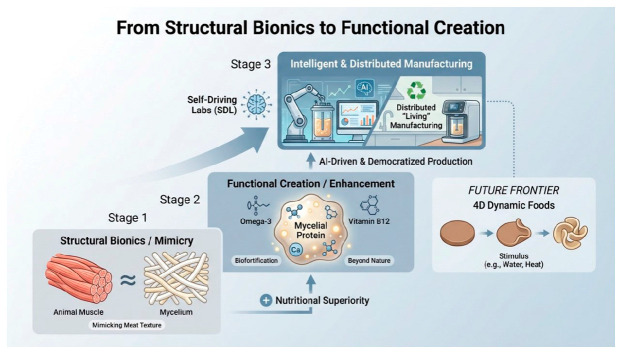
Evolutionary pathways from structural mimicry to functional innovation in alternative protein production. The diagram outlines a three-stage developmental framework: Stage 1 focuses on structural bionics, using mycelial protein to replicate the texture of animal muscle; Stage 2 advances to functional enhancement, incorporating nutrient biofortification (e.g., omega-3, vitamin B12) and 4D dynamic foods that respond to external stimuli; Stage 3 envisions the intelligent, distributed manufacturing era, enabled by self-driving labs, AI-driven production, and democratized systems for scalable and customized protein creation.

AI-driven design enables the precise identification of genetic targets for microbial engineering. This computational capability is synergistically combined with CRISPR/Cas9 gene editing, which directly reprograms cellular metabolism. For instance, blocking ethanol synthesis and gluconeogenesis pathways can engineer industrial strains with enhanced protein yield and reduced carbon emissions [57]. Commercially viable fungal proteins, exemplified by the *F. venenatum* A3/5 strain used in Quorn™, can be optimized through these advanced biotechnologies. Looking forward, the integration of predictive biology with advanced fermentation processes is poised to unlock the full potential of microbes as multifunctional living materials, enabling the co-production of protein, texture, and health-promoting compounds in a single integrated process [58]. Transient expression of CRISPR components facilitates precise genomic modifications to develop superior strains [59]. The resulting engineered microorganisms enable production of “smart foods” that address growing demographic demands through nutritional profiles surpassing conventional meat, coupled with reduced production costs and improved environmental sustainability. Furthermore, Self-Driving Laboratories (SDLs) represent a paradigm shift by integrating fully automated experimentation with artificial intelligence. These systems predict experimental outcomes and autonomously determine optimal research sequences, dramatically accelerating the development cycle of advanced food solutions [60]. Concurrently, it is essential to establish clear ethical guidelines and implement rigorous quality control protocols to ensure the responsible development of smart food technologies. This signifies a shift in focus from “meat replacement” to “optimizing nutrition”, ushering in a new chapter for personalized health foods (Figure 2).

### 5.2. Navigating Regulation and Acceptance for Microbial Foods

As microbial strategies evolve from utilizing native structures to designing novel functionalities, their regulatory and consumer acceptance pathways diverge significantly, shaped by their technological origins [61].

Mycelium-based products, rooted in traditional fermentation, typically align with established categories for novel foods, potentially streamlining approval as seen with Quorn™. Consumer perception is often favorable within the “natural fermentation” narrative. In contrast, ingredients derived from extensive genetic reprogramming face a more complex regulatory landscape, requiring comprehensive safety assessments for allergenicity, toxicity, and nutritional equivalence as per frameworks like EU Regulation 2015/2283 [61,62]. Consumer acceptance here is a major hurdle, heavily influenced by perceptions of “genetic engineering” and trust in institutions, as highlighted in systematic reviews of analogous technologies [63]. Transparent communication of benefits alongside rigorous safety validation is therefore paramount.

Thus, a nuanced understanding of these trajectories is essential. Stakeholders must proactively engage with regulators to develop appropriate frameworks [63] and invest in consumer education to build trust, ensuring that microbial innovations can responsibly reach the market.

### 5.3. Manufacturing Transformation: From Centralized Factories to Distributed “Living” Platforms

Traditional livestock production faces severe sustainability challenges, including substantial greenhouse gas emissions (accounting for approximately 18% of global anthropogenic emissions) [64], inadequate protein supply, uneven geographical distribution, and ecological disruptions caused by intensive farming practices. To address these issues, identifying viable alternative solutions requires approaches that can simultaneously enhance food security and alleviate pressures on global sustainability. Microorganisms demonstrate remarkable potential in transforming food production systems and enabling resource circularity. Research indicates that greenhouse gas emissions from food loss and waste (FLW), a consequence of unsustainable linear food systems, can be mitigated by converting food waste into nutrient sources for sustainable microalgal cultivation. This approach enhances both the technical efficiency and environmental performance of microbial lipid production [65]. Gmoser et al. demonstrated that fungal solid-state fermentation of stale bread and brewer’s spent grain significantly improved the nutritional profile, increasing dietary fiber; minerals such as copper, iron, and zinc; vitamin E; and vitamin D_2_ (reaching 0.89 μg/g dry weight). Their findings underscore the potential of using fungal-converted agricultural side streams as a sustainable strategy to reduce food waste and supplement protein supplies [66]. To effectively bridge biotechnology with societal and industrial applications, scholars advocate for interdisciplinary research infrastructure (RI) to overcome critical bioprocess scaling challenges, an essential step toward achieving sustainable, bio-based global manufacturing [67]. Edible fungal proteins serve as a prime example of this integration. By leveraging advances in industrial biotechnology, fermentation, metabolic engineering, and synthetic biology, supported by data analysis tools like multi-omics and predictive metabolic modeling, key resource utilization can be intelligently optimized to enhance microbial production systems. When combined with RI, these capabilities facilitate the establishment of nutrient-rich, recyclable, and location-independent “living” manufacturing platforms. Such systems directly address the geographical limitations and ecological disruptions inherent in traditional livestock production models. This distributed, localized, and resource-circular production model holds the potential to fundamentally restructure food supply chains, enhancing their resilience and sustainability.

### 5.4. Dynamic Response Materials and 4D Foods: Timed Changes That Give Food “Life”

4D printing technology was first conceptualized by Professor Skylar Tibbits at the Massachusetts Institute of Technology (MIT) in 2013 [68]. This concept describes 3D-printed structures fabricated from smart materials that undergo programmed physical or chemical transformations over time in response to external stimuli, such as temperature, moisture, pH, light, or ultraviolet radiation [69]. Since its inception, 4D printing technology has attracted considerable interest across academia and industry pioneered its application in the food sector, being among the first to explore its potential [70]. The distinctive advantage of 4D printing lies in its ability to produce 3D-printed foods that undergo dynamic changes in color [71,72], shape and size [73], and flavor [74] over time. These dynamic transformations offer unique opportunities to create novel sensory experiences, thereby redefining perceptions of conventional food processing and deepening human-food engagement.

**Figure 3:**
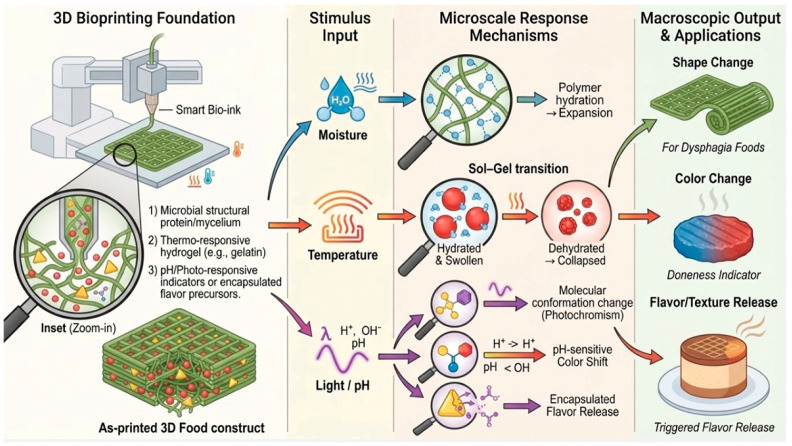
Mechanism and application potential of 4D food printing with stimulus-responsive materials. This schematic outlines the workflow from 3D printing to 4D dynamic transformation: (1) printing with smart bio-inks containing microbial polymers, thermo-responsive hydrogels, and photo/pH-sensitive compounds; (2) application of external stimuli such as moisture, temperature, or light/pH changes; (3) stimuli-triggered microscale responses, including polymer swelling, sol–gel transitions, and molecular release; (4) macroscopic transformations enabling shape-shifting, color change for indication, and controlled flavor/aroma release. Programmable microbial materials serve as “living inks” that integrate structural design with dynamic functionality.

While the application of microbial proteins in 4D printing remains unexplored, their programmable nature aligns closely with the demand for “smart materials” in 4D printing, undoubtedly representing a highly promising interdisciplinary research direction for the future. Smart materials, also termed stimulus-responsive materials, are fundamental to successful 4D food printing (4DFP). These materials alter their physical or structural properties (e.g., shape, color, texture, or flavor), and may undergo chemical changes when exposed to external stimuli such as moisture, heat, or light [75]. They can be inherently present in the food matrix or incorporated as external additives to achieve targeted transformations [76]. 4D food printing (4DFP) facilitates the creation of personalized foods incorporating specific bioactive compounds, which can be tailored to address individual nutritional requirements. For example, targeted bioactive ingredients can be integrated to manage particular health conditions, such as probiotics for gut microbiota regulation or antioxidants for anti-aging benefits [77]. Additionally, antimicrobial compounds and dietary fibers derived from lactic acid bacteria represent another promising category of functional ingredients applicable in 4DFP [13]. Xiao et al. developed dysphagia-suitable foods via 4D food printing, utilizing white mushroom powder and soy protein isolate as raw materials. The resulting products not only maintain structural integrity but also further reduce swallowing risks through dynamic structural adaptation, such as controlled hydration and expansion upon ingestion [14]. Liu et al. formulated a 3D-printable material by incorporating oyster mushroom processing byproducts with functional polysaccharides, producing structures that retained structural integrity throughout storage. Sensory analysis demonstrated significantly superior ratings for both “ease of swallowing” and “visual appeal” compared to conventional pureed food formulations. Regarding the application of microbial proteins in 4D food printing (4DFP), no studies have yet documented their use in producing meat analogs via this technology, a gap presumably owing to the field’s emergent nature. Given the growing importance of microbial protein in future food systems, its integration with 4DFP represents a promising yet underexplored frontier with significant potential to reshape food manufacturing (Figure 3).

## 6. Conclusions

Based on this review, we conclude that microorganisms serve as a pivotal driver for developing next-generation alternative proteins, offering enhanced functionality, improved sustainability, and more engaging consumer experiences. Their unique advantages include the ability to self-assemble into meat-like microstructures, efficiently convert industrial and agricultural byproducts into biomass, and undergo precision engineering via gene-editing tools such as CRISPR/Cas9. As “living structural materials”, microorganisms provide a foundational platform for the alternative protein industry. We delineate three core microbial strategies for protein structuring: solid-state fermentation as the most mature and widely adopted method; bacterial cellulose scaffolding characterized by nanoscale 3D fibrous networks and synthetic biology-enhanced precision; and synthetic biology-enabled precision design for customized production of complex proteins and novel food ingredients. These strategies require deep convergence across food, materials, and data sciences to transcend meat simulation and advance toward true food creation, ultimately paving the way for a more sustainable, healthy, and resilient food system. These frontier directions collectively sketch a vision: future foods will be not merely a source of nutrition, but “intelligent living systems” that converge biological design, precision manufacturing, and dynamic interaction.

## Data Availability

No data was used for the research described in the article.
